# Value of intra- and peritumoral ultrasound radiomics for predicting axillary lymph node burden in breast cancer

**DOI:** 10.3389/fonc.2025.1674922

**Published:** 2026-01-14

**Authors:** Mo-Han Hao, Fan Zhang, Cong Zhang, Naijing Shi, Weina Mu

**Affiliations:** 1 Department of Ultrasound Medicine, Baoding First Central Hospital, Baoding, China; 2 Department of Postgraduate, Chengde Medical University, Chengde, China; 3 Department of Urology, Baoding First Central Hospital, Baoding, China; 4 Department of Radiology, Baoding First Central Hospital, Baoding, China

**Keywords:** breast cancer, axillary lymph node burden, radiomics, ultrasound, peritumoral region

## Abstract

**Objective:**

To evaluate ultrasound-based radiomic features, derived from both intratumoral and peritumoral regions, for noninvasive preoperative prediction of axillary lymph node(ALN) burden in breast cancer.

**Methods:**

This retrospective study analyzed data from 300 pathologically confirmed breast cancer patients undergoing preoperative ultrasound. The cohort was randomly divided into a training set (n = 210) and a testing set (n = 90) at a 7∶3 ratio. Primary tumor regions of interest (ROIs) were manually delineated on preoperative ultrasound images using ITK-SNAP. Peritumoral ROIs were generated by radially expanding the intratumoral ROI by 2mm, 3mm, and 4mm. A comprehensive set of radiomic features was extracted from each ROI, with feature selection via LASSO based methods. Six machine-learning classifiers were trained on intratumoral features to identify the optimal algorithm. Using this algorithm, we built: (1) A radiomics model based solely on intratumoral or peritumoral features. (2) Combined models incorporating intratumoral and peritumoral features at each expansion margin (2mm, 3mm, and 4mm). The best-performing radiomics model was then integrated with significant clinical and conventional imaging variables to construct a composite nomogram. Model discrimination was evaluated by area under the receiver operating characteristic curve (AUC), calibration was assessed via calibration curves, and clinical utility was appraised using decision curve analysis (DCA). Model interpretability was facilitated through Shapley additive explanation (SHAP) values and visualized in a nomogram.

**Results:**

A Random Forest classifier applied to combined intratumoral and 3mm peritumoral features yielded the highest AUCs (training set: 0.825; testing set: 0.746). Multivariable logistic regression identified lesion location and ultrasonographic axillary lymph node status as independent clinical predictors (p<0.05). The integrated nomogram—combining these clinical factors with the optimal radiomics signature—demonstrated superior performance (training AUC: 0.906; testing AUC: 0.818). DCA confirmed that the combined model conferred the greatest net clinical benefit across a range of threshold probabilities, and calibration curves indicated excellent agreement between predicted and observed probabilities.

**Discussion:**

A composite model integrating intratumoral and 3mm peritumoral ultrasound radiomic features with key clinical and imaging variables enables accurate, noninvasive preoperative prediction of ALN burden in breast cancer. This approach may serve as a valuable decision support tool to guide individualized surgical planning.

## 1 Introduction

Breast cancer is the most frequently diagnosed malignancy among women worldwide and remains a leading cause of cancer-related mortality (1). According to GLOBOCAN 2022 estimates, both the incidence and mortality of breast cancer have continued to rise globally (2). In the diagnostic and therapeutic pathway for breast cancer, accurate preoperative assessment of axillary lymph node status is critical for clinical staging, treatment planning, and prognostic stratification, and it constitutes a key determinant of long-term survival outcomes (3).

The National Comprehensive Cancer Network (NCCN) guidelines recommend that, for patients with clinical T1–2 disease who have not received neoadjuvant chemotherapy and are candidates for breast-conserving surgery followed by whole-breast irradiation, low axillary lymph node burden (≤2 metastatic nodes (4)) may obviate the need for axillary lymph node dissection, whereas high burden (>3 metastatic nodes) typically warrants combined systemic therapy and axillary lymph node dissection (5). Consequently, imaging objectives have evolved from simply determining the presence or absence of nodal metastasis to the quantification of nodal tumor load (6). Preoperative quantification of axillary lymph node burden is therefore pivotal for tailoring surgical approach and minimizing postoperative complications (7). Notably, approximately 70% of early-stage breast cancer patients are ultimately found to have no nodal metastasis on postoperative pathology, suggesting that current invasive assessments may contribute to overtreatment (8). Hence, there is a pressing clinical need to develop noninvasive methods for accurately identifying axillary lymph node burden.

Ultrasound is routinely employed in this context due to its safety profile, cost-effectiveness, and real-time imaging capability (9). Conventional ultrasound assessment relies on subjective evaluation of primary tumor morphology and alterations in nodal architecture (6), but its diagnostic specificity remains limited and heavily dependent on the operator’s experience.

Radiomics—a high-throughput approach that extracts large numbers of quantitative, high-dimensional features from medical images—offers a noninvasive means of characterizing tumor heterogeneity (10). Prior radiomic investigations into breast cancer axillary lymph node status have predominantly focused on intratumoral features. However, emerging evidence indicates that the peritumoral region harbors critical biological information that may drive tumor progression, underscoring the tumor microenvironment as a potential therapeutic target (11). Most studies to date, however, lack a systematic comparison of different machine-learning classifiers, limiting the selection and optimization of the most effective predictive models (12).

In this study, we extracted both intratumoral and peritumoral radiomic features and constructed predictive models using multiple classification algorithms. We then identified the best-performing machine-learning algorithm and integrated its radiomic signature with clinicopathological variables to build a composite model, with the goal of enhancing the accuracy and robustness of noninvasive preoperative axillary lymph node burden assessment in breast cancer and providing potential for noninvasive preoperative assessment of breast cancer axillary lymph node burden.

## 2 Materials and methods

### 2.1 Study cohort

This retrospective study was designed to develop a preoperative prediction model. Accordingly, patient selection and outcome definition followed a two-stage process. This retrospective study included consecutive patients with primary breast cancer who underwent preoperative ultrasound and had core-needle biopsy (CNB) at the First Central Hospital of Baoding between January 2023 and December 2024. From an initial screen of 418 patients, 300 were enrolled based on the following preoperative criteria: (1) female patients aged >18 years; (2) complete histopathological confirmation of breast carcinoma by CNB; (3) an intent to undergo axillary surgery (SLNB or ALND), which ensured the future availability of the outcome data; (4) solitary breast lesion without distant metastasis; and (5) ultrasound imaging performed within two weeks before surgery with adequate image quality. Exclusion criteria comprised: (1) any neoadjuvant or other oncologic treatment administered prior to ultrasound; and (2) incomplete or unavailable clinical histopathological data, or suboptimal depiction of the lesion on ultrasound.

**Figure 1:**
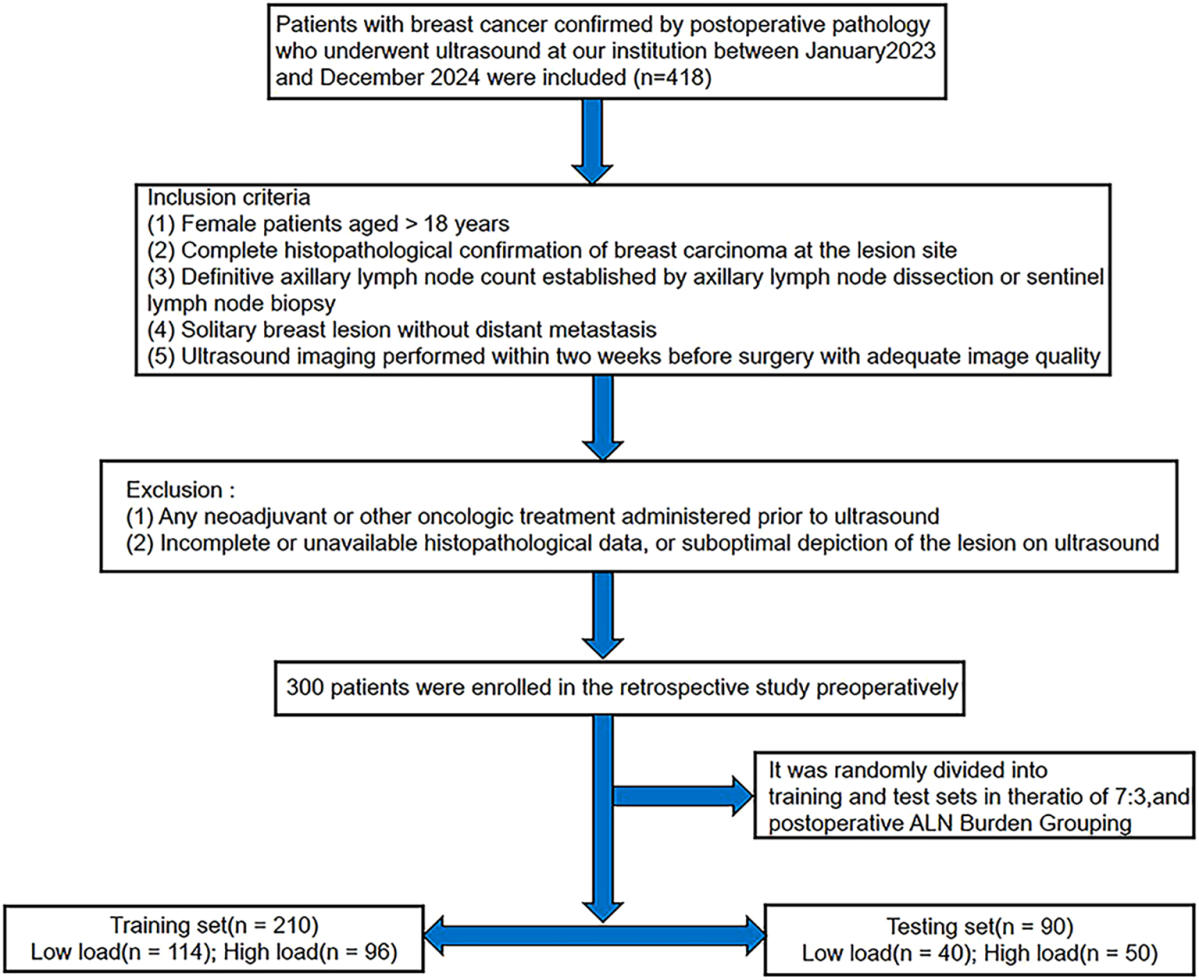
Flowchart of participant enrollment based on inclusion and exclusion criteria.

Following surgery, the outcome variable (axillary lymph node burden) was defined based on the final histopathology of the axillary surgical specimens. patients were stratified by postoperative nodal burden into low-load (≤2 metastatic nodes; n=154) and high-load (>2 metastatic nodes; n=146) groups. The cohort was then randomly allocated (7∶3) into a training set (n=210) and a testing set (n=90). The study subject flowchart is displayed in **Figure 1**.

### 2.2 Clinical data collection

Demographic and clinical variables were retrieved from the institutional pathology database for all 300 patients, with core indicators including age at diagnosis, menopausal status, family history of breast cancer, and other basic parameters.

### 2.3 Histopathological assessment

Histopathological data were obtained from percutaneous core-needle biopsy reports. It is noteworthy that CNB data, rather than surgical pathology, were intentionally utilized in this study to align with the objective of developing a preoperative predictive model. This approach ensures that all input variables, including histopathological characteristics, are available prior to surgery, thereby maintaining temporal consistency with the preoperative ultrasound imaging and reflecting real-world clinical decision-making scenarios. Variables included histologic grade (I, II, or III), tumor type, human epidermal growth factor receptor-2 (HER-2) status, estrogen receptor (ER) status, progesterone receptor (PR) status, Ki-67 proliferation index, and molecular subtype. Immunohistochemical assays defined ER and PR positivity as ≥1% of tumor-cell nuclei staining, and a Ki-67 cutoff of 14% was applied as per prior literature (13).

### 2.4 Ultrasound equipment and protocol

All patients underwent ultrasound scanning within two weeks prior to surgery using a Philips EPIQ7 color Doppler system (Philips Healthcare, Andover, MA, USA) equipped with an L12–5 linear-array transducer operating at 8–12MHz. Patients were positioned supine with both arms raised above or alongside the head to fully expose the breasts and axillae. Comprehensive sweeps of both transverse and longitudinal planes were performed. The image demonstrating the maximal lesion long-axis diameter in the standard plane was saved for subsequent radiomic analysis.

### 2.5 Two-dimensional ultrasound feature analysis

Ultrasound images were independently reviewed by two attending radiologists with 5 and 8 years of experience, respectively, under blinded conditions. Assessed ultrasound features included lesion size, location, and axillary ultrasound status. axillary ultrasound status was considered positive for nodal involvement if any of the following criteria were met: cortical thickness >3mm, long-to-short axis ratio <2, cortical-to-medullary thickness ratio >1, or absence of the fatty hilum (14). Discrepancies between the two readers were resolved by consensus with a third senior radiologist.

### 2.6 Ultrasound radiomic analysis

#### 2.6.1 ROI delineation and feature extraction

Ultrasound images were exported in NIfTI format (.nii.gz) and imported into ITK-SNAP. Two breast-imaging specialists (each with 5 years of experience), blinded to clinical and histopathological data, manually delineated the intratumoral region of interest (ROI) on the two-dimensional ultrasound slice depicting the lesion’s maximum long-axis dimension. Based on this intratumoral ROI, peritumoral ROIs were generated by isotropically expanding the contour by 2mm, 3mm, and 4mm; the original intratumoral ROI was then subtracted to yield pure peritumoral ROIs at each margin. All ROIs were saved in NIfTI format. A senior radiologist subsequently reviewed and approved all segmentations.

Radiomic features of the intratumoral, peritumoral, and combined intratumoral + peritumoral ROIs were automatically extracted using the open-source PyRadiomics package, including shape features, first-order statistical features, texture features, and wavelet features.

#### 2.6.2 Feature selection, dimensionality reduction, and model development

All radiomic features were standardized via Z-score normalization. Univariate analyses (independent-samples t test or Mann–Whitney U test, as appropriate) and Pearson correlation analysis were employed to preselect features associated with axillary lymph node burden. Subsequently, least absolute shrinkage and selection operator (LASSO) regression with ten-fold cross-validation was applied to identify the optimal regularization parameter (λ) and to select the subset of nonzero-coefficient features most predictive of axillary lymph node status.

Six machine-learning classifiers—Extreme Gradient Boosting (XGBoost), Random Forest, Support Vector Machine (SVM), K-nearest neighbors (KNN), Extra Trees, and Multilayer Perceptron (MLP)—were each trained using the intratumoral radiomic signature to determine the best-performing algorithm. This optimal algorithm was then used to develop seven radiomic signatures: one intratumoral model, three peritumoral models (2mm, 3mm, and 4mm), and three combined intratumoral+peritumoral models (2mm/3mm/4mm).

Clinical and conventional ultrasound features were subjected to univariate and multivariate logistic regression to identify independent predictors. A clinical model was constructed from those variables reaching statistical significance. Finally, the radiomic signature (“radiomics score”) was combined with significant clinical predictors in a multivariable logistic regression framework to construct the integrative (combined) model.

#### 2.6.3 Feature selection, dimensionality reduction, and model development

Model discrimination was assessed by plotting receiver operating characteristic (ROC) curves and calculating the area under the curve (AUC), sensitivity, specificity, and overall accuracy for each model. Pairwise comparisons of AUCs were conducted using DeLong’s test to determine statistically significant differences in performance. Calibration of the combined model was evaluated using calibration curves to verify agreement between predicted probabilities and observed outcomes. Clinical utility was quantified via decision curve analysis (DCA), which estimates the net benefit across a range of threshold probabilities, thus demonstrating the potential of each model to guide clinical decision-making.

### 2.7 Statistical analysis

Statistical analyses were performed using SPSS version 26.0 and R software. Continuous variables conforming to a normal distribution are presented as mean±standard deviation (x¯±s) and compared between groups using the independent-samples t test; non-normally distributed data are expressed as median (interquartile range, M [Q1, Q3]) and compared using the Mann–Whitney U test. Categorical variables are summarized as counts (n) and compared using the chi-square test (χ²) or Fisher’s exact test, as appropriate. Ordinal variables were analyzed with the Wilcoxon rank-sum test. A two-sided P value <0.05 was considered statistically significant.

### 2.8 Model visualization

**Figure 2:**
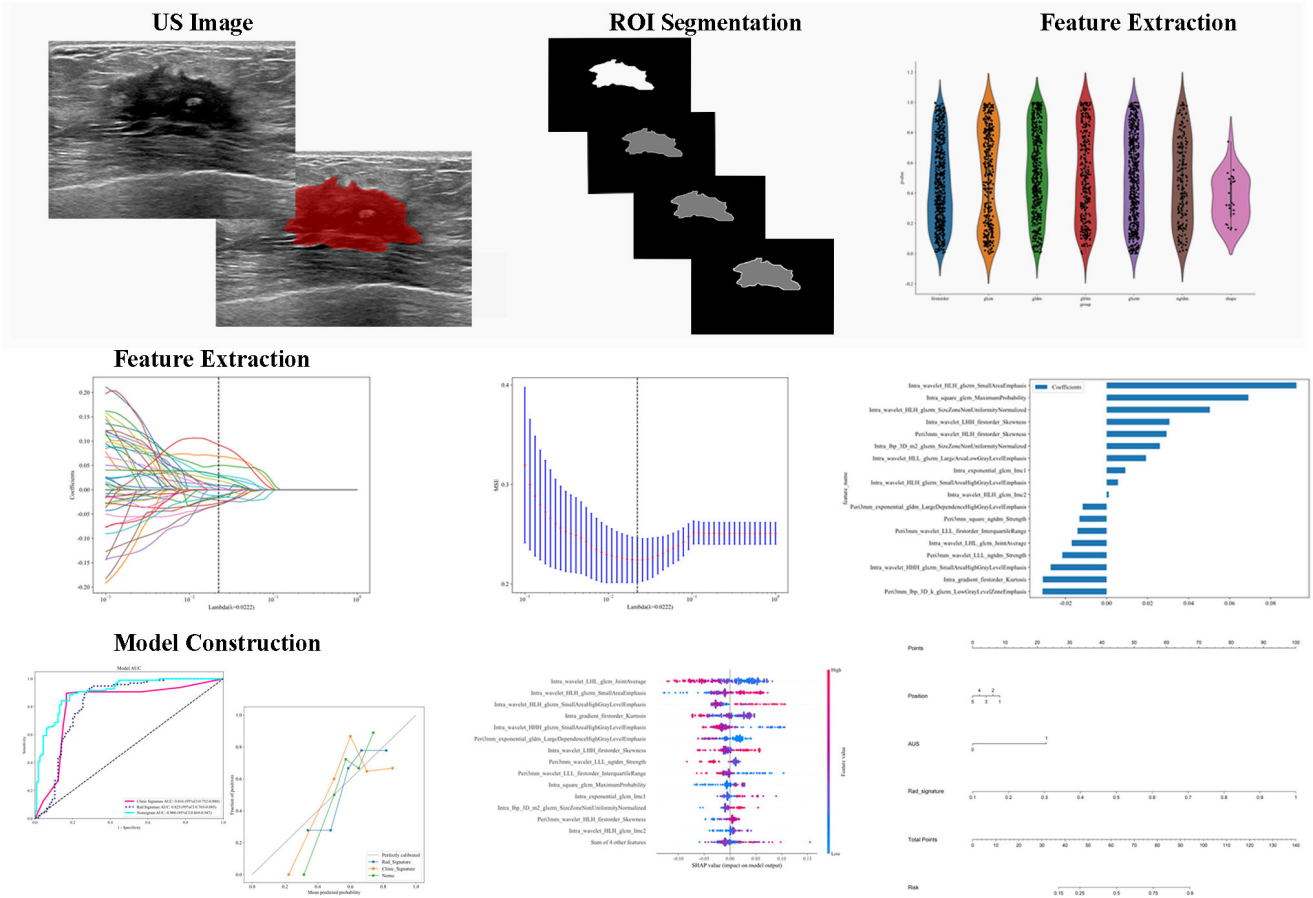
The workflow of radiomics analysis in this study.

The optimal intratumoral+peritumoral radiomics model was coupled with Shapley Additive Explanations (SHAP) to visualize and interpret the radiomic signature. Global SHAP analyses quantified and ranked the consistent contributions of individual radiomic features to the prediction of axillary lymph node status, thereby elucidating the association patterns between features and the outcome. Additionally, SHAP force-plot visualizations for individual patients illustrated the patient-specific predictive mechanisms underlying nodal metastasis. Independently significant clinical predictors were then combined with the radiomics signature to construct a nomogram, providing an integrated, user-friendly visualization of the overall predictive model. The workflow of radiomics analysis in this study is presented in **Figure 2**.

## 3 Results

### 3.1 Baseline characteristics

A total of 300 patients were included based on preoperative criteria. After surgical intervention and postoperative pathological assessment, they were stratified into low axillary lymph node burden (n=154) and high axillary lymph node burden (n=146) groups. The cohort was randomly assigned (7∶3) to a training set (n=210) and a testing set (n=90). Comparison of clinicopathological parameters and ultrasound features between the training and testing sets revealed no significant differences (all P>0.05).Refer to **Table 1**, confirming the comparability of the two cohorts for subsequent modeling.

**Table 1:** Comparison of clinicopathological and imaging features between the training and testing sets.

Variables	Total (n = 300)	Test (n = 90)	Train (n =210)	Statistic	P
Age, Mean ± SD	57.10 ± 11.94	57.31 ± 12.32	57.01 ± 11.81	t=0.20	0.842
Volume, Mean ± SD	15. 11 ± 16.42	14.54 ± 12.83	15.35 ± 17.76	t=-0.39	0.697
Menopausal status, n(%)				χ²=1.03	0.311
Premenopausal	113 (37.67)	30 (33.33)	83 (39.52)		
Postmenopausal	187 (62.33)	60 (66.67)	127 (60.48)		
Family history, n(%)				χ²=0.24	0.624
Yes	17 (5.67)	6 (6.67)	11 (5.24)		
No	283 (94.33)	84 (93.33)	199 (94.76)		
Histologic grade, n(%)				χ²=0.55	0.76
I	63 (21.00)	19 (21. 11)	44 (20.95)		
II	190 (63.33)	59 (65.56)	131 (62.38)		
III	47 (15.67)	12 (13.33)	35 (16.67)		
Her2, n(%)				χ²=0.97	0.325
Negative	143 (47.67)	39 (43.33)	104 (49.52)		
Positive	157 (52.33)	51 (56.67)	106 (50.48)		
Er, n(%)				χ²=1.97	0.16
Negative	96 (32.00)	34 (37.78)	62 (29.52)		
Positive	204 (68.00)	56 (62.22)	148 (70.48)		
Pr, n(%)				χ²=1.10	0.294
Negative	97 (32.33)	33 (36.67)	64 (30.48)		
Positive	203 (67.67)	57 (63.33)	146 (69.52)		
Ki 67, n(%)				χ²=0.76	0.385
Low expression	94 (31.33)	25 (27.78)	69 (32.86)		
High expression	206 (68.67)	65 (72.22)	141 (67. 14)		
Location, n(%)				χ²=0.78	0.941
Upper- inner quadrant	98 (32.67)	32 (35.56)	66 (31.43)		
Lower- inner quadrant	54 (18.00)	15 (16.67)	39 (18.57)		
Upper- outer quadrant	84 (28.00)	23 (25.56)	61 (29.05)		
Lower- outer quadrant	45 (15.00)	14 (15.56)	31 (14.76)		
Central zone	19 (6.33)	6 (6.67)	13 (6. 19)		
Pathological type, n(%)				χ²=0.46	0.499
Invasive	250 (83.33)	73 (81. 11)	177 (84.29)		
Non-invasive	50 (16.67)	17 (18.89)	33 (15.71)		
Molecular subtype, n(%)				χ²=1.59	0.661
Luminal A	64 (21.33)	18 (20.00)	46 (21.90)		
Luminal B	151 (50.33)	43 (47.78)	108 (51.43)		
HER2‐overexpressing	60 (20.00)	22 (24.44)	38 (18. 10)		
Triple‐negative	25 (8.33)	7 (7.78)	18 (8.57)		
Diameter, n(%)				χ²=0.17	0.682
≤2cm	122 (40.67)	35 (38.89)	87 (41.43)		
>2cm	178 (59.33)	55 (61. 11)	123 (58.57)		
Axillary lymph node status,n(%)				χ²=3.30	0.069
Negative	144 (48.00)	36 (40.00)	108 (51.43)		
Positive	156 (52.00)	54 (60.00)	102 (48.57)		

t: t-test, χ²: Chi-square test.

SD, standard deviation.

### 3.2 Optimal ultrasound radiomics model development and visualization

#### 3.2.1 Model construction

**Figure 3:**
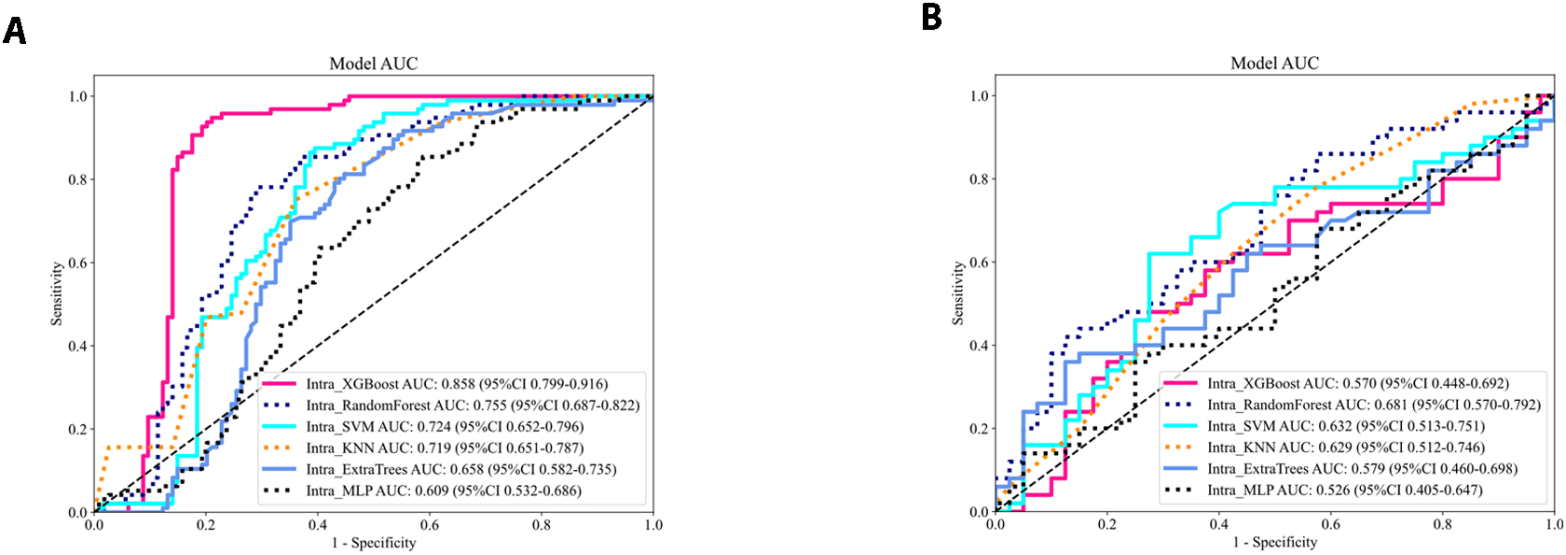
**(A, B)** Comparison of diagnostic performance of intratumoral models using different machine learning ROC curves in the training set **(A)** and the testing set **(B)**.

For each patient, 1–562 radiomic features were extracted from the intratumoral and peritumoral ROIs at 2 mm, 3 mm, and 4 mm. Following univariate filtering (t test and Pearson correlation) and LASSO-based dimensionality reduction, corresponding radiomic signatures were established. Six classifiers—XGBoost, Random Forest (RF), support vector machine (SVM), k-nearest neighbors (KNN), Extra Trees, and multilayer perceptron (MLP)—were trained on the intratumoral signature, yielding AUCs in the training set of 0.853, 0.755, 0.724, 0.719, 0.658, and 0.609, respectively, and validation-set AUCs of 0.570, 0.681, 0.632, 0.629, 0.579, and 0.526. **Figures 3A, B** shows The Random Forest classifier achieved the highest validation AUC (0.681) and was selected as the optimal algorithm. Its performance metrics were: training AUC 0.755 (95% CI: 0.687 – 0.822) and validation AUC 0.681 (95% CI: 0.570 – 0.792). This Random Forest algorithm was then applied to build all seven radiomic models (intratumoral alone; peritumoral at 2 mm, 3 mm, 4 mm; and combined intratumoral + peritumoral at 2 mm, 3 mm, 4 mm).

**Figure 4:**
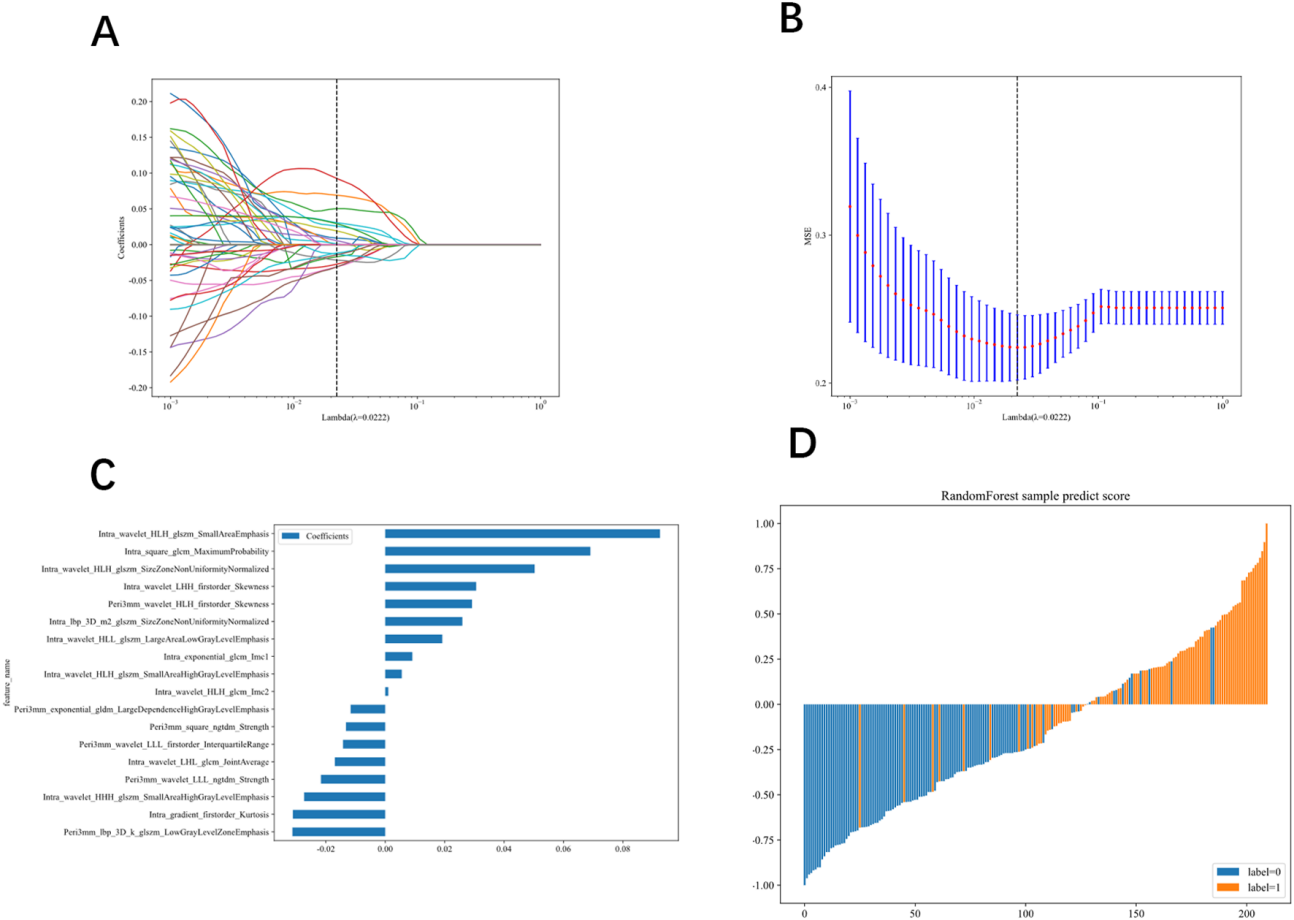
**(A–C)** Parameter selection via LASSO regression, showing optimal regularization parameter λ **(A)**. Coefficient profiles of radiomic features versus log(λ),highlighting nonzero coefficients **(B)**. Feature importance weights for the radiomic signature **(C)**. Histogram of sample prediction probabilities generated by the Random Forest model **(D)**.

**Figure 5:**
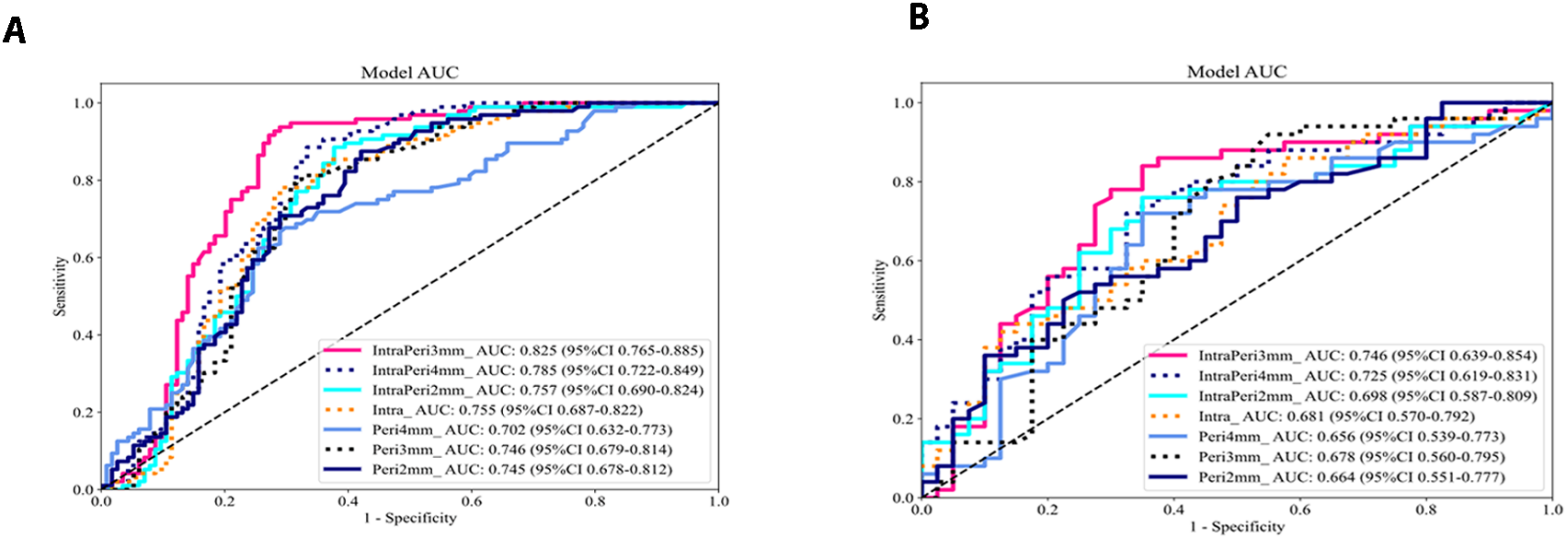
**(A, B)** Comparison of ROC Curves for Intratumoral, Peritumoral, and Combined Models in the training set **(A)** and the testing set **(B)**.

ROC analysis demonstrated that the intratumoral + 3 mm peritumoral model achieved the highest AUC and was therefore designated the optimal radiomic model. After feature selection, 18 features remained (12 intratumoral, 6 peritumoral)(see **Figures 4A-D**), which were used to calculate the radiomics signature. A detailed description of these features, including their intuitive interpretation, is provided in “**Supplementary Table S1**”.In the training and validation cohorts, this signature yielded AUCs of 0.825 (95% CI: 0.765 – 0.885) and 0.746 (95% CI: 0.639 – 0.854)(see **Figures 5A, B**), sensitivities of 0.937 and 0.840, and specificities of 0.711 and 0.650, respectively. Refer to **Table 2**.

**Table 2:** Comparison of performance of intratumoral, peritumoral, and combined models in the training and testing sets.

Model_name	AUC	95% CI	Accuracy	Sensitivity	Specificity	PPV	NPV	Precision	F1
Training set									
IntraPeri3mm_	0.825	0.7646 - 0.8845	0.814	0.937	0.711	0.732	0.931	0.732	0.822
IntraPeri4mm_	0.785	0.7217 - 0.8490	0.767	0.885	0.667	0.691	0.874	0.691	0.776
IntraPeri2mm_	0.757	0.6901 - 0.8239	0.743	0.885	0.623	0.664	0.866	0.664	0.759
Intra_	0.755	0.6873 - 0.8219	0.743	0.781	0.711	0.694	0.794	0.694	0.735
Peri4mm_	0.702	0.6319 - 0.7730	0.69	0.677	0.702	0.657	0.721	0.657	0.667
Peri3mm_	0.746	0.6786 - 0.8138	0.738	0.812	0.675	0.678	0.811	0.678	0.739
Peri2mm_	0.745	0.6781 - 0.8122	0.714	0.875	0.579	0.636	0.846	0.636	0.737
Testing set									
IntraPeri3mm_	0.746	0.6388 - 0.8542	0.756	0.84	0.65	0.75	0.765	0.75	0.792
IntraPeri4mm_	0.725	0.6189 - 0.8311	0.7	0.72	0.675	0.735	0.659	0.735	0.727
IntraPeri2mm_	0.698	0.5872 - 0.8088	0.711	0.76	0.65	0.731	0.684	0.731	0.745
Intra_	0.681	0.5699 - 0.7921	0.622	0.42	0.875	0.808	0.547	0.808	0.553
Peri4mm_	0.656	0.5386 - 0.7734	0.689	0.72	0.65	0.72	0.65	0.72	0.72
Peri3mm_	0.677	0.5601 - 0.7949	0.711	0.92	0.45	0.676	0.818	0.676	0.78
Peri2mm_	0.664	0.5509 - 0.7766	0.622	0.5	0.775	0.735	0.554	0.735	0.595

Delong’s test indicated that, in the training set, the AUC of the intratumoral + 3 mm peritumoral model was significantly higher than that of the 4 mm peritumoral model (P = 0.006), and the intratumoral + 4 mm peritumoral model also outperformed the 4 mm peritumoral model (P = 0.029). In the testing set, no pairwise differences reached statistical significance (P > 0.05), possibly due to the smaller sample size.

#### 3.2.2 Model visualization

**Figure 6:**
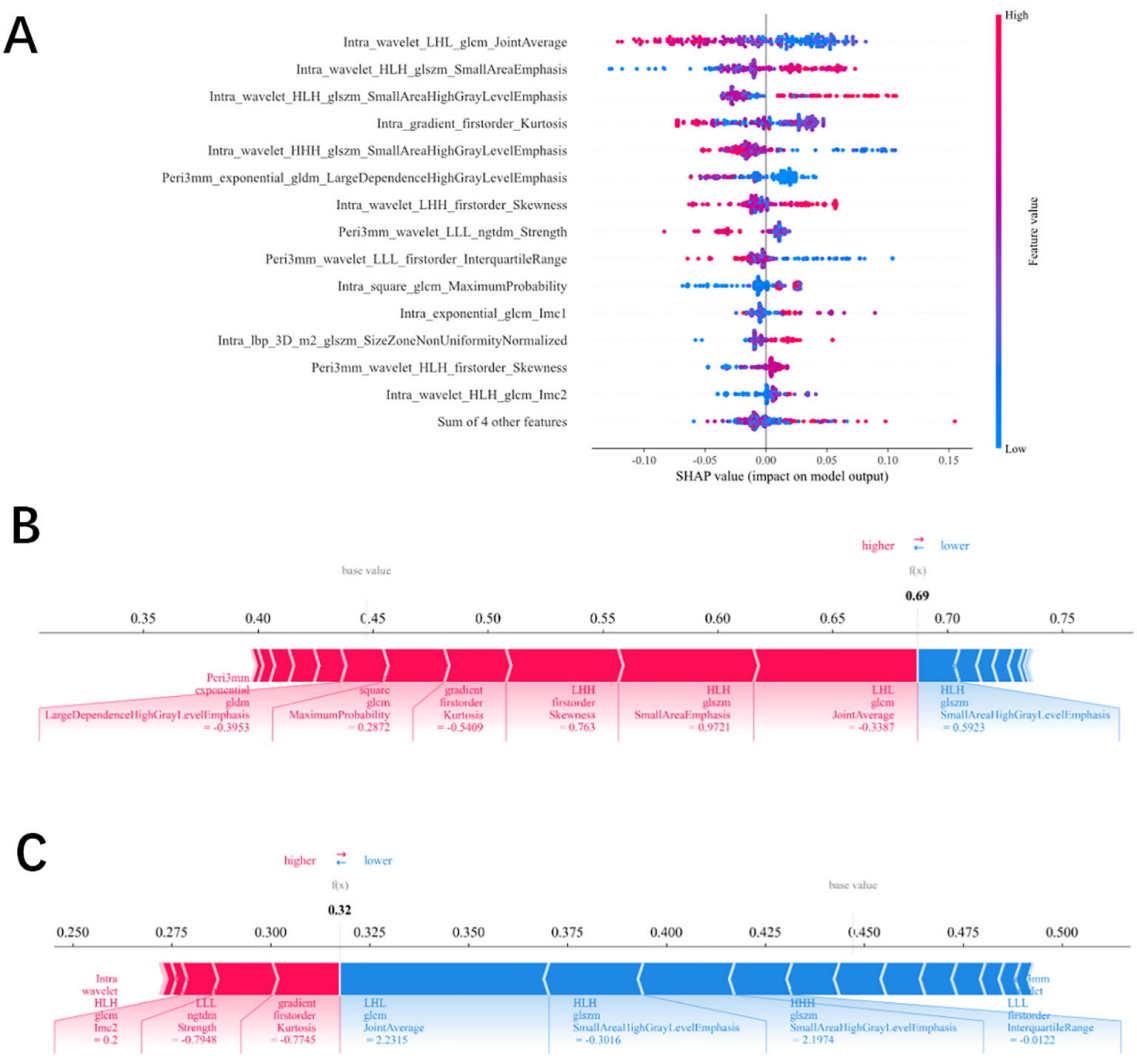
**(A–C)** Single-Sample SHAP Analysis **(A)**. SHAP force-plot explaining how the radiomics model differentiates ALN status (final predicted value=0.687, above the baseline 0.447, predicting low burden) **(B)**. SHAP force-plot explaining how the model differentiates ALN status for another patient (final predicted value=0.317, below the baseline 0.447, predicting high burden) **(C)**.

The optimal radiomics model developed in this study was visualized using SHAP values to display the contribution of each feature (see **Figure 6A**).

In the swarm plot, dense scatter points map the strength of each variable’s impact on the prediction; each point represents an individual sample, and the color gradient (blue→red) reflects the continuous distribution of feature values from low to high. As shown, Intra_wavelet_LHL_glcm_JointAverage is the feature with the largest contribution to predicting axillary lymph node burden. As its value decreases, the SHAP value increases, indicating a higher predicted probability of nodal burden.

The waterfall plot decomposes the SHAP values for a single sample(see **Figures 6B, C**), using the model’s expected value (E[f(X)]) as the baseline and visualizing the incremental contribution of each feature in sequence. Red arrows indicate an increased risk of low axillary lymph node burden, while blue arrows indicate a decreased risk.

### 3.3 Nomogram construction and performance evaluation

**Figure 7:**
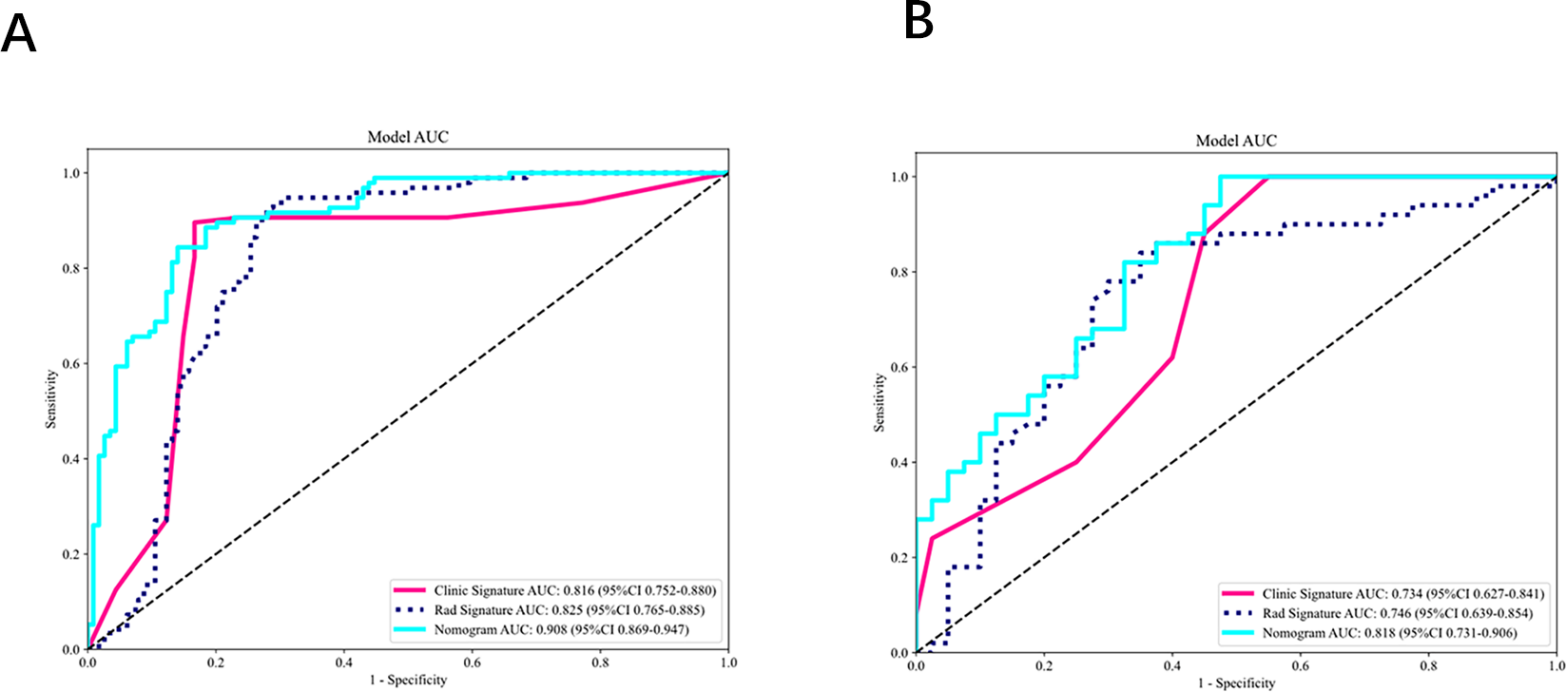
**(A, B)** ROC Curves Comparing the Radiomics Model, Clinical Model, and Combined Model in the training set **(A)** and the testing set **(B)**.

On univariate and multivariate analyses of conventional ultrasound features and clinicopathological variables, axillary ultrasound status and lesion location emerged as independent risk factors for axillary lymph node burden (P<0.05).Refer to **Table 3**. These two predictors were used to develop the clinical model. ROC analysis yielded AUCs of 0.816 (95% CI: 0.752–0.880) in the training set and 0.734 (95% CI: 0.627–0.841) in the testing set(see **Figures 7A, B**).

**Table 3:** Univariate and multivariate logistic regression analysis of ultrasound features.

Variables	Univariate		Multivariate	
	OR(95%CI)	P value	OR(95%CI)	P value
Family history	0.777(0.614-0.982)	0.077		
Histologic grade	0.932(0.834-1.042)	0.297		
Her2	0.963(0.700-1.326)	0.846		
Er	0.897(0.685-1.177)	0.511		
Pr	0.872(0.664-1.146)	0.408		
Ki 67	0.785(0.594-1.038)	0.153		
Pathological type	0.941(0.530-1.669)	0.862		
Molecular subtype	0.923(0.836-1.020)	0.187		
Axillary lymph node status	1.684(1.202-2.358)	0.011	3.728(2.358-5.894)	0
Menopausal status	0.868(0.647-1.163)	0.425		
Tumor location	0.901(0.829-0.980)	0.041	0.724(0.641-0.818)	0
Age	0.998(0.994-1.001)	0.293		
Volume	0.992(0.981-1.002)	0.166		
Diameter	0.757(0.561-1.021)	0.127		

**Figure 8:**
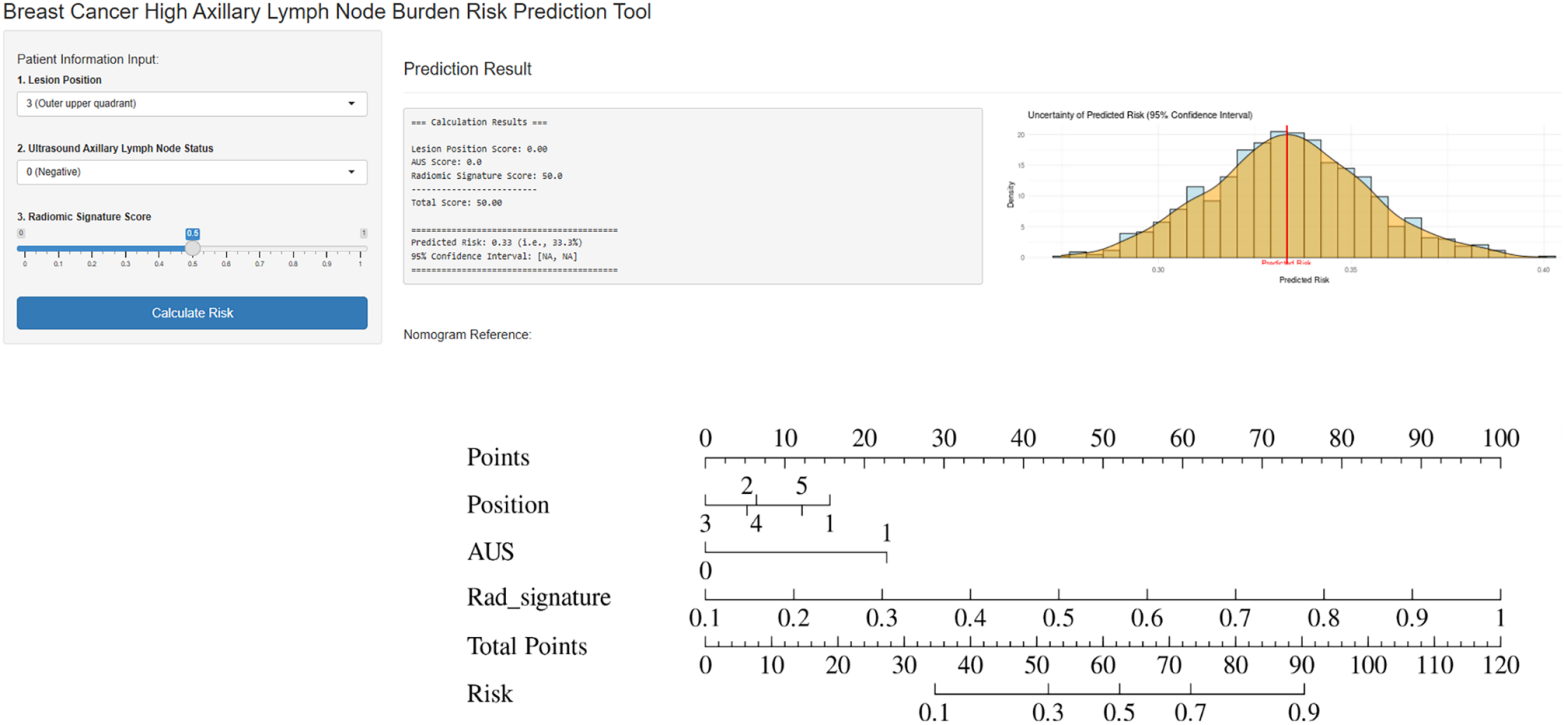
An online nomogram for predicting high ALND has been established, which includes the following three parameters: axillary lymph node status, lesion position, and radiomic signature score. By accessing the link [https://my-nomogram-app.shinyapps.io/my-nomogram-app/], the demonstration shows that when the lesion is located in the outer upper quadrant, the axillary lymph node status is negative, and the radiomic signature score is 0.5, the nomogram predicts a 33.3% probability of high ALND.

**Figure 9:**
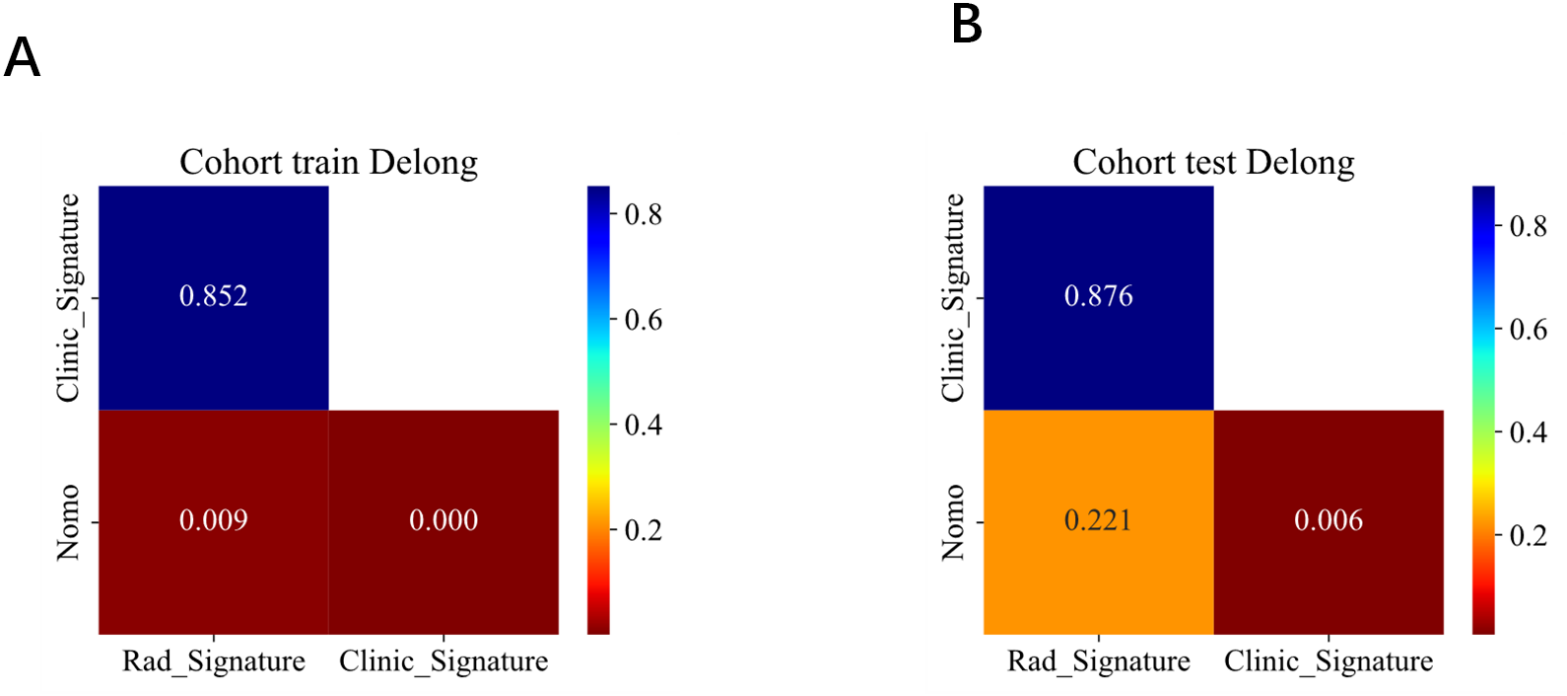
**(A, B)** Comparison of model performance between the training **(A)** and testing sets **(B)**.

A combined model was then constructed by integrating the independent clinical risk factors with the radiomics signature. In the training set, the combined model achieved an AUC of 0.908, accuracy of 0.852, and specificity of 0.860; in the testing set, it yielded an AUC of 0.818, accuracy of 0.789, and specificity of 0.525 (see **Table 4**). A nomogram was generated to visualize the combined model, and its interactive online implementation (available at: https://my-nomogram-app.shinyapps.io/my-nomogram-app/) is presented in **Figure 8**, which includes the original nomogram in the lower-right corner for verification and reference. Delong’s test demonstrated that, in the training set, the combined model significantly outperformed both the intratumoral+3mm peritumoral radiomics model (P=0.009) and the clinical model alone (P<0.001). In the testing set, the combined model also showed a statistically significant improvement over the clinical model (P=0.006) (see **Figures 9A, B**).

**Table 4:** Comparison of performance of the radiomics model, clinical model, and combined model.

Model_name	AUC	95% CI	Accuracy	Sensitivity	Specificity	PPV	NPV	Precision	F1
Training set									
Clinic Signature	0.816	0.7517 - 0.8802	0.862	0.896	0.833	0.819	0.905	0.819	0.856
Rad Signature	0.825	0.7646 - 0.8845	0.814	0.937	0.711	0.732	0.931	0.732	0.822
Nomogram	0.908	0.8688 - 0.9472	0.852	0.844	0.86	0.835	0.867	0.835	0.839
Testing set									
Clinic Signature	0.734	0.6270 - 0.8410	0.756	1	0.45	0.694	1	0.694	0.82
Rad Signature	0.746	0.6388 - 0.8542	0.756	0.84	0.65	0.75	0.765	0.75	0.792
Nomogram	0.818	0.7313 - 0.9057	0.789	1	0.525	0.725	1	0.725	0.84

**Figure 10:**
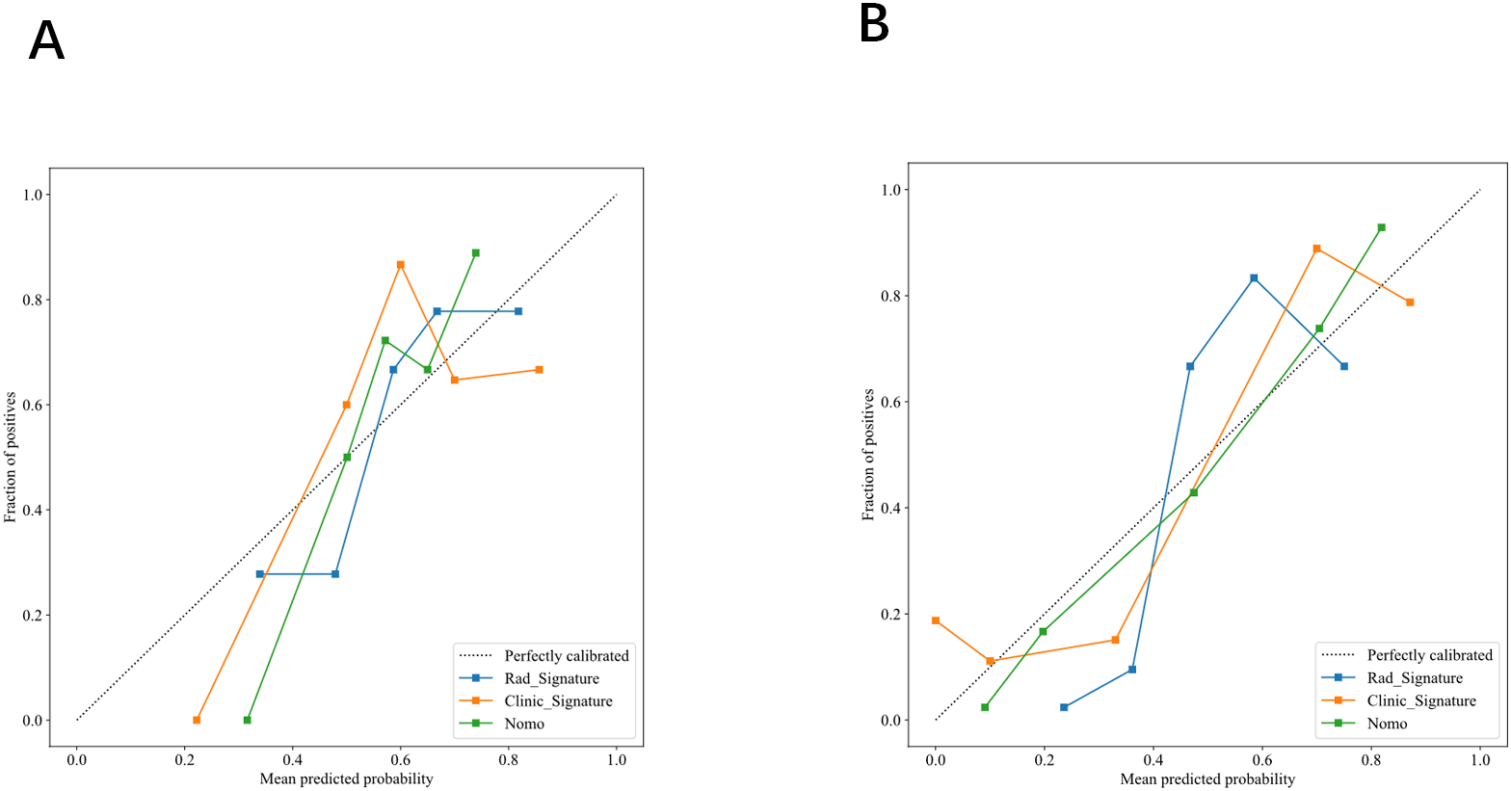
**(A, B)** Calibration curves for the training **(A)** and testing sets **(B)**.

**Figure 11:**
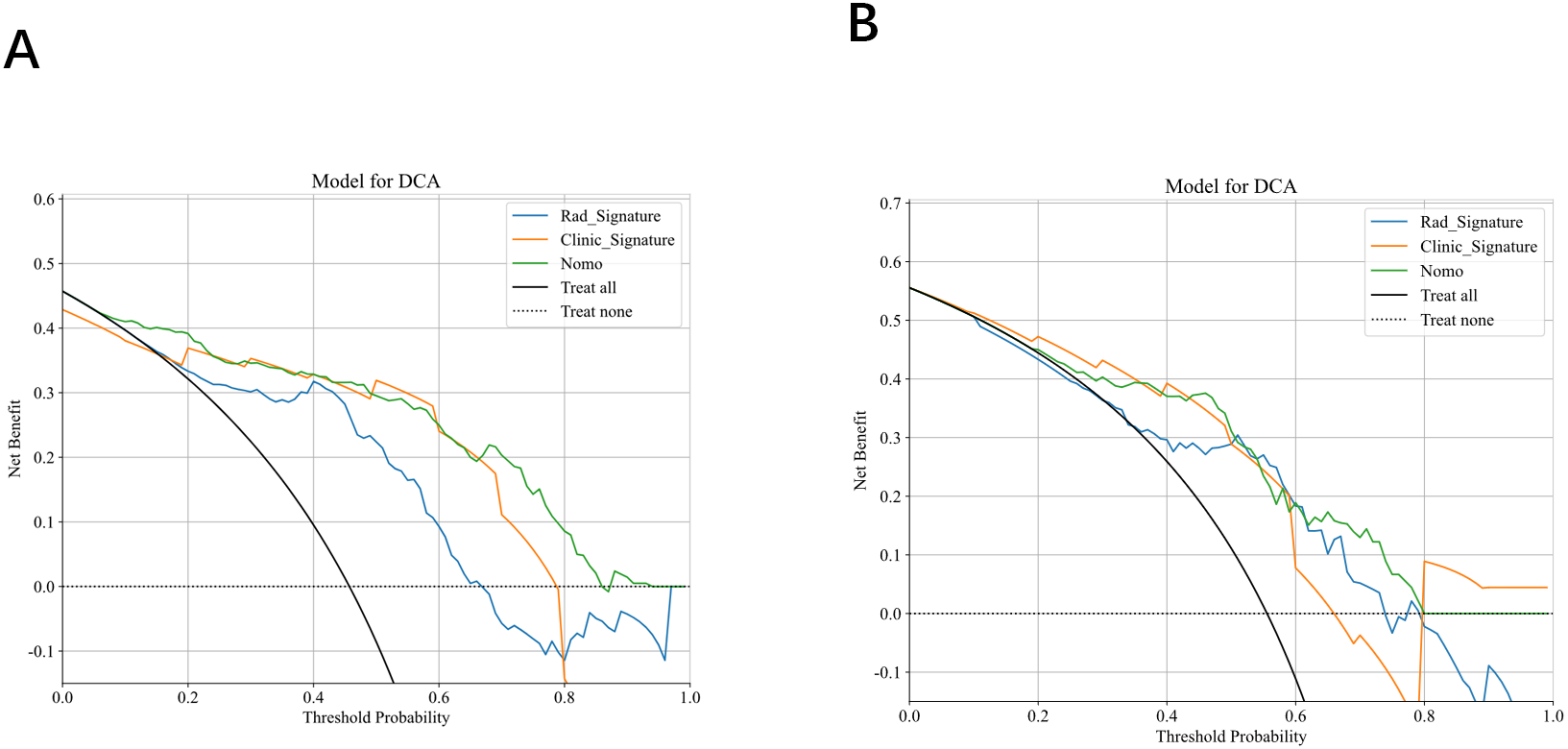
**(A, B)** Decision curve analysis for the training **(A)** and testing sets **(B)**.

Calibration curves indicated excellent agreement between predicted probabilities and observed outcomes for axillary lymph node burden in both the training and validation cohorts(see **Figures 10A, B**). Decision‐curve analysis revealed that, across a wide range of threshold probabilities, the combined model provided greater net clinical benefit than the clinical or radiomics models alone. Specifically, in the training set, the combined model was superior when the threshold probability ranged from 0.07–0.25 and 0.67–0.87; in the testing set, its advantage was most pronounced at thresholds of 0.45–0.58 and 0.63–0.80 (see **Figures 11A, B**).

## 4 Discussion

Accurate assessment of axillary lymph node status is pivotal in clinical practice for disease staging, individualized surgical decision-making, and prognostic evaluation. Patients with high nodal burden typically require axillary lymph node dissection and systemic therapy, whereas those with low burden may avoid axillary lymph node dissection and thereby reduce the risk of procedure-related complications (15). Notably, the biological underpinnings of axillary lymph node burden are closely linked to the tumor microenvironment. Breast tumors consist of malignant epithelial cells and stromal components, the latter mediating peritumoral extracellular matrix remodeling. Given these clinical imperatives and pathophysiological mechanisms, the development of a noninvasive, preoperative method to evaluate axillary lymph node burden holds substantial value for precise triage and optimized therapeutic selection. In this study, we employed six machine-learning algorithms to construct intratumoral radiomics models, identified the optimal classifier, and extended it to peritumoral (2mm, 3mm, and 4mm) and combined intratumoral+peritumoral models. We then integrated independent clinical predictors with the superior radiomics signature to build a combined model, aiming to enable noninvasive prediction of breast cancer axillary lymph node burden and provide more precise decision-support for clinicians. Furthermore, our model is built exclusively on preoperatively available data, including CNB-based pathology, which reinforces its practical utility for decision-making prior to any surgical intervention.

Previous investigations have demonstrated that tumor diameter, histologic grade, molecular subtype, and pathologic type variably influence breast cancer axillary lymph node burden (16–18). In our cohort, these factors did not differ significantly between high- and low-burden groups, which may reflect the cohort’s broad clinical diversity, limitations in sample size, or population characteristics. Earlier studies have explored the predictive value of ultrasound-detected axillary lymph node positivity for nodal burden in breast cancer (19). Our current work corroborates that sonographic axillary lymph node status is an independent predictor: metastatic tumor cells infiltrate lymphatic sinuses, inducing diffuse cortical thickening, medullary invasion, and displacement or obliteration of the fatty hilum (20), with aberrant cell proliferation leading to nodal enlargement and morphological distortion (19). Consequently, suspicious ultrasound features correlate positively with the extent of tumor invasion and axillary lymph node burden.

Hwang et al. (21) reported superior survival in patients with tumors located in the upper-outer quadrant, attributing this to earlier detection and clearance of axillary metastases, whereas lesions in the inner-lower quadrant more frequently metastasize to the less accessible internal mammary nodes. Consistent with these findings, our study identified primary tumor location as an independent determinant of axillary lymph node burden. The clinical model constructed from these factors achieved an AUC of 0.816 in the training set and 0.734 in the testing set, reflecting moderate predictive performance. Importantly, compared to prior clinicopathologic models, our approach enables noninvasive preoperative evaluation of axillary lymph node status.

Ultrasound radiomics has demonstrated significant value in differentiating benign from malignant breast lesions, identifying molecular subtypes, and predicting response to neoadjuvant therapy (22).

In the field of preoperative axillary lymph node status prediction, Gao etal. developed a two-dimensional ultrasound-based radiomics model for predicting axillary lymph node metastasis, achieving an AUC of 0.723 in the validation cohort (23). Although their methodology parallels ours, their study was limited to invasive carcinoma, whereas our research encompassed a broader spectrum of pathologies—including invasive lobular carcinoma, invasive carcinoma of no special type, and ductal carcinoma in situ—thereby enhancing the model’s generalizability. Given that the progression of aggressive malignancies is often accompanied by stromal proliferation or interstitial infiltration, the imaging characteristics of the peritumoral tissue harbor critical biological information (24). Samantha etal. combined peritumoral features to construct a composite model for predicting axillary lymph node status in clinically node-negative breast cancer patients, yielding an AUC of 0.86 (25). Dong etal. found that diagnosis based solely on peritumoral tissue was insufficient (26), a conclusion our study corroborates: the combined model’s AUC consistently exceeded that of models containing only intratumoral or only peritumoral features. As the spatial pattern extends from intratumoral to peritumoral regions, these models more comprehensively capture tumor heterogeneity and invasiveness (27). Zhang etal. reported that a diagnostic model integrating intratumoral and peritumoral radiomic features with clinical variables accurately predicted axillary lymph node status, with the tumor+3mm margin model performing best (28).

Consistent with our findings, the intratumoral+3mm peritumoral model maximally assessed axillary lymph node status, whereas prior studies have noted practical limitations in defining a 5mm peritumoral margin (29). For superficially or deeply located lesions, a 5mm expansion often extends beyond the breast parenchyma boundary, introducing non-target tissue and compromising accuracy. Distinctively, our study evaluated six different machine-learning algorithms for intratumoral modeling and ultimately selected Random Forest—an algorithm known for robustness and resistance to overfitting, with documented high diagnostic performance (30)—further enhancing predictive efficacy.

To enhance clinical applicability, we integrated the radiomics signature with independent clinical predictors to develop a combined model, achieving AUCs of 0.908 and 0.818 in the training and validation cohorts, respectively, alongside high clinical utility. We employed both SHAP and a nomogram to visualize the model: SHAP provides an intuitive depiction of the Random Forest decision process by computing Shapley values from cooperative game theory, thereby quantifying each feature’s marginal contribution to the model’s output. This elucidates feature importance at both cohort and individual levels, bolstering interpretability and clinician trust (31). Prior studies have validated SHAP’s value in explicating machine-learning models (32). In our results, the SHAP-identified top contributing feature “Intra_wavelet_LHL_glcm_JointAverage”, a wavelet-based co-occurrence matrix feature. It captures subtle textural patterns on ultrasound, imperceptible in routine assessment, by measuring the average gray-level intensity of pixel pairs. Biologically, lower values of this metric indicate a more disrupted tumor microarchitecture, which is associated with a higher ALN burden. The nomogram offers a straightforward visualization of the combined model’s prediction workflow, incorporating easily obtainable preoperative variables (tumor location and sonographic axillary lymph node status), thus enabling noninvasive, individualized prediction of axillary lymph node burden. The integrated application of SHAP and the nomogram establishes complementary interpretative frameworks for both imaging and clinical features. This synergy enhances model transparency, credibility, and clinical utility while providing novel methodological insights for future research.

This study has several limitations. First, its retrospective, single-center design may introduce selection bias. Second, the sample size constitutes the primary constraint; an ‘Events Per Variable’ (EPV) analysis confirmed a suboptimal ratio, which inherently elevates overfitting risk as reflected in the performance gap between training and test sets. Although mitigation strategies were employed, this fundamental limitation persists. Although we employed mitigation strategies—such as dataset partitioning, rigorous feature selection, and the use of algorithms with anti-overfitting properties—to reduce this risk, nevertheless, this fundamental limitation remains an important consideration for the interpretation of our results. Furthermore, only two-dimensional ultrasound images were analyzed; three-dimensional, elastography, and contrast-enhanced ultrasound modalities were not included. Finally, we implemented traditional machine-learning methods without exploring the recently advanced deep-learning techniques that offer improved data processing and generalization capabilities.

It is important to note that the broad clinical diversity of our cohort stemmed from deliberate design choices to enhance generalizability. This included a pragmatic application of inclusion criteria, such as accepting borderline-quality images assessed by consensus and defining “solitary lesion” in a clinically relevant manner. Furthermore, our inclusive approach resulted in a spectrum of pathological types and molecular subtypes. To verify that this diversity did not unduly influence our core findings, a sensitivity analysis was performed wherein the optimal radiomics model was retrained on a homogeneous invasive ductal carcinoma subgroup (n=152). The model’s performance remained robust (AUC = 0.736) and was not significantly different from its performance on the full cohort (AUC = 0.746; DeLong’s p = 0.818), confirming the stability of our radiomic signature across the cohort’s clinical and pathological spectrum. (See **Supplementary Figures 1A, B**).

In summary, future studies using larger, prospective, multi-center datasets are essential to further validate and refine our model and to robustly assess its clinical generalizability, and evaluate deep-learning approaches for axillary lymph node burden prediction.

## 5 Towards clinical application

To bridge the gap between our findings and clinical application, a clear translation pathway has been initiated. We have developed an online nomogram-based calculator to facilitate initial use. Our immediate focus is to engineer a semi-automated image analysis module, eliminating the dependency on manual feature extraction. Subsequent prospective validation will then be conducted to rigorously evaluate the tool’s performance and workflow efficiency within real clinical settings, paving the way for its practical adoption.

## 6 Conclusion

By applying the optimal radiomics algorithm to combined intratumoral and peritumoral ultrasound radiomic signatures alongside clinical predictors, we established a noninvasive, individualized model with robust diagnostic performance for preoperative prediction of axillary lymph node status in breast cancer patients. The incorporation of SHAP and a nomogram not only offers clear, graphical model interpretation but also enables an individualized preoperative prediction framework, thereby providing more precise guidance for clinical decision making.

## Data Availability

The raw data supporting the conclusions of this article will be made available by the authors, without undue reservation.
