# Supplementary figures and images for: Value of intra- and peritumoral ultrasound radiomics for predicting axillary lymph node burden in breast cancer
Source: Front Oncol. 2026 Jan 14;15:1674922. doi: 10.3389/fonc.2025.1674922 (PMC12847015; doi:10.3389/fonc.2025.1674922)

Supplementary Figure1A, B

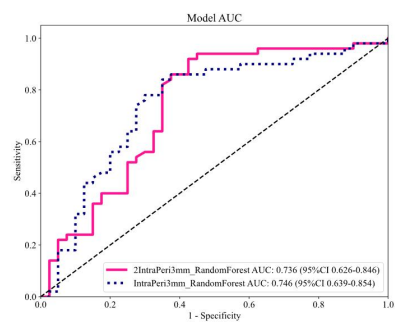

A

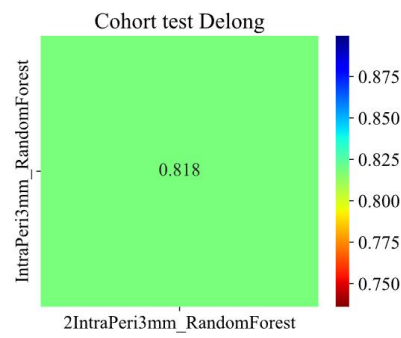

B

Supplement: Supplementary file 1 [file DataSheet1.pdf]
