# Supplementary material for: Value of intra- and peritumoral ultrasound radiomics for predicting axillary lymph node burden in breast cancer
Source: Front Oncol. 2026 Jan 14;15:1674922. doi: 10.3389/fonc.2025.1674922 (PMC12847015; doi:10.3389/fonc.2025.1674922)
Supplement: Supplementary file 2 [file DataSheet2.pdf]

Supplementary Table 1

Feature Name	Category	Simple Explanation	What a High/Low Value Might Mean
Intra_wavelet_HLH_glszm_SmallAreaEmphasis	Wavelet+ Gray Level Size Zone Matrix	Measures the emphasis on small areas within the tumor region after HLH wavelet decomposition.	A higher value may suggest microstructural heterogeneity in the tumor, associated with an increased risk of ALN burden.
Intra_square_glcm_Maximum Probability	Gray Level Co-occurrence Matrix	Represents the highest probability of pixel pairs with the same gray level within the tumor.	A higher maximum probability may suggest more uniform cell arrangement, potentially associated with a lower risk of ALN burden.
Intra_wavelet_HLH_glszm_SizeZoneNonUniformityNormalized	Wavelet+Gray Level Size Zone Matrix	Quantifies the normalized non-uniformity of zone sizes within the tumor after HLH wavelet decomposition.	Higher non-uniformity may reflect disordered tumor structure, associated with a higher ALN burden.
Intra_wavelet_HLH_firstorder_ Skewness	Wavelet+ First-Order Statistics	Describes the asymmetry of the gray-level distribution within the tumor after HLH wavelet decomposition.	Skewness in the gray-level distribution may indicate abnormal cell density, related to the potential for ALN metastasis.
Intra_lbp_3D_m2_glszm_SizeZoneNonUniformityNormalized	Local Binary Pattern+Gray Level Size Zone Matrix	Quantifies the normalized non-uniformity of zone sizes within the tumor using 3D LBP and GLSZM.	Non-uniformity in zone sizes may suggest tumor heterogeneity, associated with a higher risk of ALN burden.
Intra_wavelet_HLH_glszm_LargeAreaLowGrayLevelEmphasis	Wavelet+Gray Level Size Zone Matrix	Emphasizes large, low gray-level areas within the tumor after HLH wavelet decomposition.	Large, low gray-level areas may suggest hypoechoic regions, associated with aggressive tumor behavior and ALN burden.
Intra_exponential_glcm_Imc1	Gray Level Co-occurrence Matrix	Represents the first informational measure of correlation in the exponential GLCM of the tumor.	A higher value may suggest greater spatial irregularity of tumor cells, associated with ALN metastasis.
Intra_wavelet_HLH_glszm_SmallAreaHighGrayLevelEmphasis	Wavelet+Gray Level Size Zone Matrix	Emphasizes small, high gray-level areas within the tumor after HLH wavelet decomposition.	Small, high gray-level areas may reflect microcalcifications or dense cell clusters, potentially promoting ALN burden.
Intra_wavelet_HLH_glcm_Imc2	Wavelet+Gray Level	Represents the second informational measure of	A higher value may suggest more complex cell arrangement patterns,

	Co-occurrence Matrix	correlation in the GLCM of the tumor after HLH wavelet decomposition.	associated with the potential for ALN metastasis.
Intra_wavelet_HHH_glszm_SmallAreaHighGrayLevelEmphasis	Wavelet+Gray Level Size Zone Matrix	Emphasizes small, high gray-level areas within the tumor after HHH wavelet decomposition.	Small, high gray-level areas may reflect focal dense cell regions, associated with the potential for ALN metastasis.
Intra_gradient_firstorder_ Kurtosis	Gradient+ First-Order Statistics	Describes the 'peakedness' of the gray-level gradient distribution within the tumor.	Higher kurtosis may indicate abrupt changes in cell density, associated with ALN burden.
Intra_wavelet_LHL_glcm_JointAverage	Wavelet+ Gray Level Co-occurrence Matrix	Represents the joint average of gray-level pairs in the GLCM of the tumor after LHL wavelet decomposition.	A higher joint average may suggest a more uniform gray-level distribution, potentially associated with a lower risk of ALN burden.
Peri3mm_exponential_gldm_LargeDependenceHighGrayLevel Emphasis	Peritumoral+ Exponential +Gray Level Dependence Matrix	Emphasizes large dependence on high gray-level areas in the exponential GLDM of the 3mm peritumoral region.	Presence of large, bright, coarse-textured areas in the periphery is associated with ALN burden.
Peri3mm_square_ngtdm_ Strength	Peritumoral+ Neighboring Gray Tone Difference Matrix	Measures the strength of the gray-level distribution in the 3mm peritumoral region using square NGTDM.	Higher strength may suggest homogeneity in the peritumoral tissue, potentially associated with a lower risk of ALN burden.
Peri3mm_wavelet_LLL_ firstorder_InterquartileRange	Peritumoral+ Wavelet+ First-Order Statistics	Quantifies the interquartile range of gray levels in the 3mm peritumoral region after LLL wavelet decomposition.	A wider interquartile range may reflect heterogeneity in the peritumoral region, potentially promoting ALN burden.
Peri3mm_wavelet_LLL_ ngtdm_Strength	Peritumoral+ Wavelet+ Neighboring Gray Tone Difference Matrix	Measures the strength of the gray-level distribution in the 3mm peritumoral region using NGTDM after LLL wavelet decomposition.	Higher strength may suggest regularity in the peritumoral tissue, associated with a lower ALN burden.
Peri3mm_lbp_3D_k_glszm_LowGrayLevel ZoneEmphasis	Peritumoral+ Local Binary Pattern+Gray Level Size Zone Matrix	Emphasizes low gray-level zones in the 3mm peritumoral region using 3D LBP and GLSZM.	Low gray-level zones may reflect peritumoral edema or inflammation, associated with ALN burden.

Peri3mm_wavelet_HLH_firstorder_Skewness	Peritumoral+ Wavelet+ First-Order Statistics	Measures the skewness of the gray-level distribution in the 3mm peritumoral region after HLH wavelet decomposition.	Skewness in the peritumoral microenvironment may reflect tumor invasiveness, potentially promoting ALN burden.
