# Supplementary material for: Value of intra- and peritumoral ultrasound radiomics for predicting axillary lymph node burden in breast cancer
Source: Front Oncol. 2026 Jan 14;15:1674922. doi: 10.3389/fonc.2025.1674922 (PMC12847015; doi:10.3389/fonc.2025.1674922)
Supplement: Supplementary file 3 [file DataSheet3.pdf]

**Supplementary File 2: Reproduction Protocol for Radiomics Analysis of Breast Cancer Axillary Lymph Node Burden Prediction Model**

**Overview**

This reproduction protocol for radiomics analysis is intended to provide detailed instructions for other researchers to replicate the complete radiomics workflow of constructing the axillary lymph node (ALN) burden prediction model for breast cancer in this study. The workflow is implemented based on the open-source **OnekeyAI Platform**, and all relevant codes and configuration files have been made publicly available.

**Public code repository:** https://github.com/OnekeyAI-Platform

2. Software Environment and Dependencies

**Operating System:** Windows 10/11 is recommended.

**Environment Management:** Anaconda3 (Python 3.8+).

**Core Dependent Packages:**

pyradiomics==3.0.1

scikit-learn==1.0.2

xgboost==1.5.0

itk-snap (for ROI delineation)

**3. Data Preparation and Directory Structure**

Before running the code, organize your data according to the

following structure:

Your_Project_Directory/ # Project root directory

**Images：** Raw ultrasound images (.nii.gz format)

1.nii.gz： Raw image of Patient 1

2.nii.gz： Raw image of Patient 2

...

**masks：** Intratumoral/peritumoral segmentation masks (.nii.gz format, filenames must match those in "images")

1.nii.gz： Intratumoral + peritumoral (2mm/3mm/4mm) masks of Patient 1

2.nii.gz： Intratumoral + peritumoral (2mm/3mm/4mm) masks of Patient 2

...

**Clinical：** Clinical data table (including patient ID, labels, and key clinical indicators)

Note: The filenames in the images and masks directories must correspond one-to-one to ensure correct data matching.

**4. Step-by-Step Reproduction Workflow (Listing of Core Steps)**

Steps such as data preprocessing and feature extraction call the onekey/onekey_comp/comp9-SolutionsJ module, and nomogram construction calls the onekey/onekey_comp/comp7-Survival module. The complete paths of all modules are clearly marked in the repository.

**Step 1. Environment Configuration**

Install Anaconda3.exe. Create a virtual environment in Anaconda → activate the environment → install dependent packages.

**Step 2. ROI Delineation**

Use ITK-SNAP software to manually delineate the tumor region (intratumoral region) on the raw ultrasound images, and use its expansion function to generate 2mm, 3mm, and 4mm peritumoral regions. Store the delineated intratumoral and peritumoral mask files in the masks directory.

**Step 3. Data Preparation**

Organize the three folders (images, masks, and clinical) corresponding to patients' raw images, delineated images, and clinicopathological data respectively. Ensure that each patient's .nii.gz file is stored separately.

Steps 4 to 12 below all use onekey/onekey_comp at main · OnekeyAI-Platform/onekey .The following are partial code snippets; please refer to the above public code repository for the complete code.

**Step 4: Data Preprocessing and Validation**

**1.Execution Module**: onekey/onekey_comp/comp9-SolutionsJ

**2.Partial Code Snippet**:

```
1.images, masks = get_image_mask_from_dir(mydir, images='images', masks='masks')
```

```
# def get_images_mask(mydir):
#     images = []
#     masks = []
#     for root, dirs, files in os.walk(mydir):
#         for f in files:
```

**3.Function:** This script will automatically validate the file format and quantity matching of files in the images and masks directories to ensure that data can be read correctly.

**Step 5: Radiomic Feature Extraction**

Execution Module: onekey/onekey_comp/comp9-SolutionsJ

**1.Key Parameters:** Feature extraction uses the PyRadiomics library, and its parameters are defined by the custom_settings/exampleUS.yaml file. This study used the default parameter set in this configuration file.

**2.Output:** The script will generate a table file (rad_features.csv) containing all extracted features.

Partial Code Snippet:

```
import warnings
import pandas as pd
warnings.filterwarnings("ignore")
from onekey_algo.custom.components.Radiology import ConventionalRadiomics
if os.path.exists('results/rad_features.csv'):
```

**Step 6: Feature Engineering and Dataset Construction**

Execution Module: onekey/onekey_comp/comp9-SolutionsJ

Workflow:

1.Data Integration: Merge radiomic features with annotation data.

Partial Code Snippet:

```
from onekey_algo.custom.components.comp1 import normalize_df
data = normalize_df(combined_data, not_norm=labels, group=group_info)
data = data.dropna(axis=1)
data.describe()
```

2.Standardization: Perform Z-score standardization on the integrated features to eliminate the influence of dimension.

Partial Code Snippet:

```
combined_data = pd.merge(rad_data, label_data, on=['ID'], how='inner')
ids = combined_data['ID']
combined_data = combined_data.drop(['ID'], axis=1)
print(combined_data[labels].value_counts())
combined_data
```

**Step 7: Feature Selection**

**1.Execution Module**: onekey/onekey_comp/comp9-SolutionsJ

**2.Workflow**: A two-step method is used for feature dimensionality reduction:

a. Calculate the Pearson correlation coefficient between features and labels, and remove highly redundant features.

b. LASSO Regression Refined Selection: Use 10-fold cross-validation LASSO regression to further screen features, and select non-zero coefficient features as the features for subsequent models.**Specific Parameters**: Lasso — scikit-learn 1.7.2 documentation

**Partial Code Snippet**

```
pearson_corr = data[data['group'] == 'train'][[c for c in data.columns if c not in labels]].corr('pearson')
# kendall_corr = data[[c for c in data.columns if c not in labels]].corr('kendall')
# spearman_corr = data[[c for c in data.columns if c not in labels]].corr('spearman')
```

**Step 8. Feature Weight**

**1.Execution Module**: onekey/onekey_comp/comp9-SolutionsJ

**2.Partial Code Snippet**:

```
feat_coef = sorted(feat_coef, key=lambda x: x[1])
feat_coef_df = pd.DataFrame(feat_coef, columns=['feature_name', 'Coefficients'])
```

```
feat_coef_df.plot(x='feature_name', y='Coefficients', kind='barh')
plt.savefig(f'img/Rad_feature_weights.svg', bbox_inches = 'tight')
```

**Step 9: Model Training and Comparison**

**Execution Module**: onekey/onekey_comp/comp9-SolutionsJ

1.Randomly divide the dataset into a training set and a testing set at a ratio of 7:3.

**Partial Code Snippet**

```
import numpy as np
import onekey_algo.custom.components as okcomp
n_classes = 2
train_data = sel_data[(sel_data[group_info] == 'train')]
train_ids = ids[train_data.index]
```

2.Use the screened features to train multiple machine learning classifiers (including Random Forest, SVM, XGBoost, etc.) on the training set.

**Partial Code Snippet**

```
model_names = ['SVM', 'KNN', 'RandomForest', 'ExtraTrees', 'XGBoost', 'LightGBM', 'MLP', 'LR']
models = okcomp.comp1.create_clf_model(model_names)

model_names = list(models.keys())
```

3.Evaluate the performance of each model on the testing set. As stated in the paper, the **Random Forest** model was selected as the final model due to its optimal and stable performance.

**Step 10: Model Result Visualization (e.g., AUC, ROC Curve)**

**Execution Module**: onekey/onekey_comp/comp9-SolutionsJ

**Partial Code Snippet**:

```
sel_model = model_names

for pred_score, label in zip(pred_scores, labels):
    pred_test_scores = []
    for sm in sel_model:
```

**Step 11: Model Interpretation and Visualization**

SHAP Analysis:

Partial Code

```
from onekey_algo.custom.viz.shap_clf import get_explainer, get_shap_value
os.makedirs('img', exist_ok=True)
explainer, model, features, shap_values = get_explainer(model_path, feature_path)
shap_value = get_shap_value(shap_values, analysis_label=analysis_label)
```

```
shap.plots.beeswarm(shap_value, show=False, max_display=15)
plt.savefig(f"img/feature_shap_value_distribution.svg", bbox_inches='tight')
plt.show()
```

Function: Run the SHAP analysis script to generate a feature importance summary plot (e.g., beeswarm plot) to explain the decision basis of the final model (Random Forest).

**2.Nomogram Construction:**

Execution Module: onekey/onekey_comp/comp7-Survival

Partial Code Snippet:

```
nomogram.nomogram(df, duration='duration', result='result',

            columns=['position', 'aus','Rad_signature'],
            survs=[36, 60],
            surv_names=['3-year Survival', '5-year Survival'],

            height=5800)
```

Function: Combine significant clinical predictors with radiomic signatures to construct a nomogram for individualized prediction.

**5. Notes**

a. Ensure that all file paths are configured correctly.b. To ensure the

consistency of reproduction results, key random number seeds have been fixed in the code.c. This document is a summary workflow. For detailed parameters, function definitions, and annotations of all scripts, please refer to the actual code in the GitHub repository.

Through the above steps, we ensure that other researchers can replicate the complete workflow of this study. We sincerely invite reviewers and peer researchers to access our code repository to gain in-depth understanding of the details of the computational workflow and verify the reproducibility of this study.
